# Reprimo tissue-specific expression pattern is conserved between zebrafish and human

**DOI:** 10.1371/journal.pone.0178274

**Published:** 2017-05-31

**Authors:** Ricardo J. Figueroa, Gonzalo Carrasco-Avino, Ignacio A. Wichmann, Martin Lange, Gareth I. Owen, Arndt F. Siekmann, Alejandro H. Corvalán, Juan C. Opazo, Julio D. Amigo

**Affiliations:** 1 Departamento de Fisiología, Facultad de Ciencias Biológicas, Pontificia Universidad Católica de Chile, Santiago, Chile; 2 Advanced Center for Chronic Diseases (ACCDiS), Santiago, Chile; 3 Pathology Department, Hospital Clínico Universidad de Chile, Santiago, Chile; 4 Departamento de Oncología y Hematología, Facultad de Medicina, Pontificia Universidad Católica de Chile, Santiago, Chile; 5 Max Planck Institute for Molecular Biomedicine, Muenster, Germany; 6 Millennium Institute on Immunology and Immunotherapy, Santiago, Chile; 7 Center UC for Investigation in Oncology (CITO), Pontificia Universidad Católica de Chile, Santiago, Chile; 8 Instituto de Ciencias Ambientales y Evolutivas, Facultad de Ciencias, Universidad Austral de Chile, Valdivia, Chile; University of Lausanne, SWITZERLAND

## Abstract

*Reprimo* (*RPRM*), a member of the *RPRM* gene family, is a tumor-suppressor gene involved in the regulation of the p53-mediated cell cycle arrest at G2/M. *RPRM h*as been associated with malignant tumor progression and proposed as a potential biomarker for early cancer detection. However, the expression and role of *RPRM*, as well as its family, are poorly understood and their physiology is as yet unstudied. In this scenario, a model system like the zebrafish could serve to dissect the role of the *RPRM* family members *in vivo*. Phylogenetic analysis reveals that *RPRM* and *RPRML* have been differentially retained by most species throughout vertebrate evolution, yet *RPRM3* has been retained only in a small group of distantly related species, including zebrafish. Herein, we characterized the spatiotemporal expression of *RPRM* (present in zebrafish as an infraclass duplication *rprma/rprmb)*, *RPRML* and *RPRM3* in the zebrafish. By whole-mount *in situ* hybridization (WISH) and fluorescent *in situ* hybridization (FISH), we demonstrate that *rprm* (*rprma/rprmb*) and *rprml* show a similar spatiotemporal expression profile during zebrafish development. At early developmental stages *rprmb* is expressed in somites. After one day post-fertilization, *rprm* (*rprma/rprmb*) and *rprml* are expressed in the notochord, brain, blood vessels and digestive tube. On the other hand, *rprm3* shows the most unique expression profile, being expressed only in the central nervous system (CNS). We assessed the expression patterns of *RPRM* gene transcripts in adult zebrafish and human *RPRM* protein product in tissue samples by RT-qPCR and immunohistochemistry (IHC) staining, respectively. Strikingly, tissue-specific expression patterns of the *RPRM* transcripts and protein are conserved between zebrafish and humans. We propose the zebrafish as a powerful tool to elucidate the both physiological and pathological roles of the *RPRM* gene family.

## Introduction

**Fig 1:**
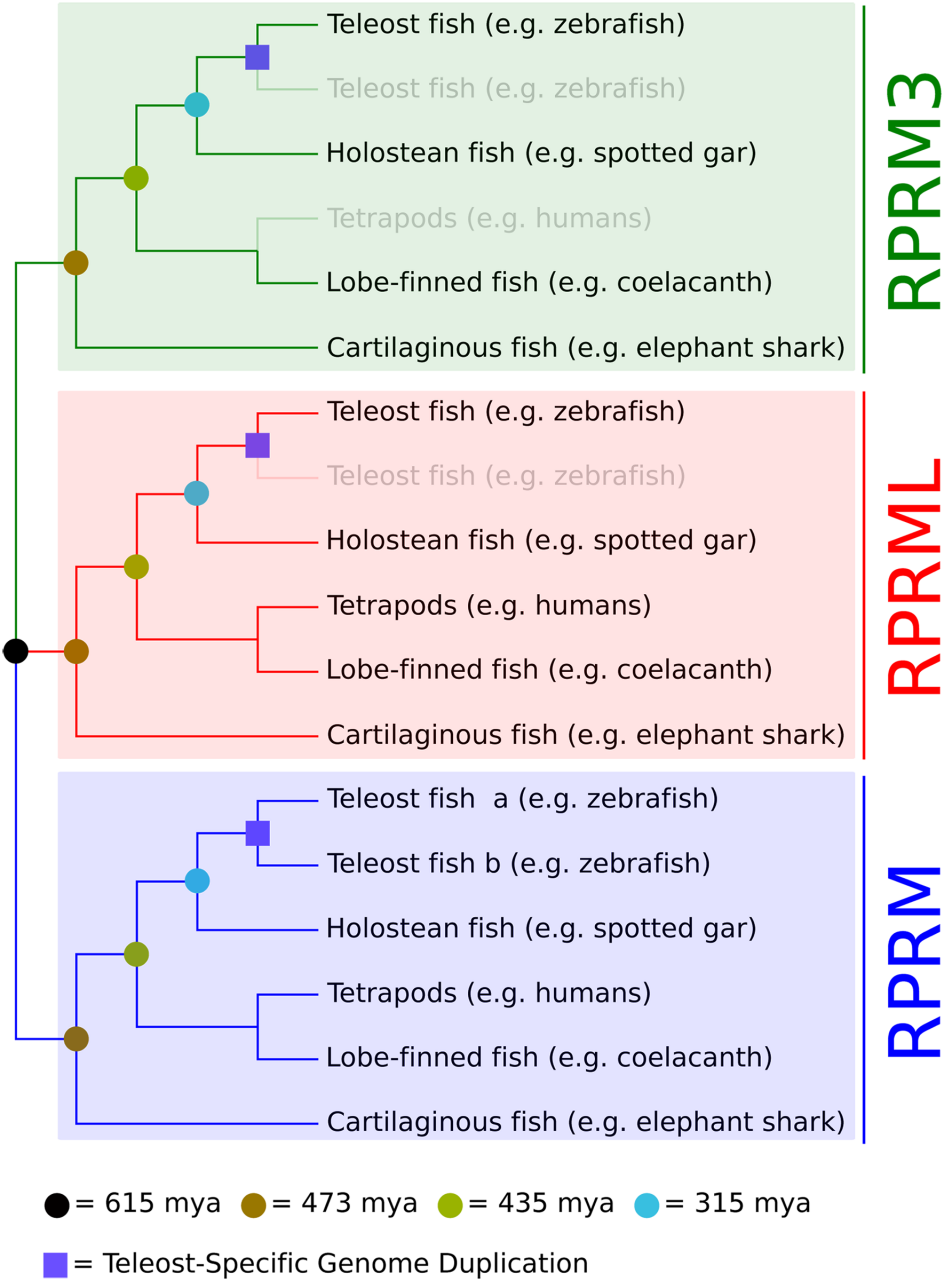
Evolutionary history of the *reprimo* gene family in gnathostome vertebrates. *Reprimo* genes (*RPRM*, *RPRML* and *RPRM3*) diversified as a product of the two rounds of whole genome duplication occurred in the last common ancestor of vertebrates. After that, they were differentially retained among the different group of vertebrates. Teleost fish (e.g. zebrafish) underwent an extra round of whole genome duplication that further expanded this group of genes, however, not all *reprimo* genes were retained in all species. Thus, teleost species retained duplicated copies only in the *RPRM* gene lineage, while in the other two (*RPRML* and *RPRM3*), only one of them was retained. Diffuse lines represent gene lineages that are not retained in actual species. Divergence time estimates were obtained from the TimeTree public database [38]. The 615 mya estimates correspond to the last common ancestor of vertebrates.

**The *Reprimo* (***RPRM)* gene family has only been identified in vertebrates and that is composed by three paralogs: *RPRM*, *RPRML* and *RPRM3* [1]. This group of genes diversified during the two rounds of whole genome duplication that occurred early in the evolution of vertebrates. Following duplication, family members were differentially retained [1]. Thus, *RPRM* and *RPRML* have been retained in most vertebrates, including humans; whereas *RPRM3* has been retained only in a small group of distantly related species [1], including the zebrafish (Fig 1). Synteny analyses further confirmed that these copies were originated as a product of whole genome duplication, as genes found up- and down-stream conserved in both zebrafish copies were, to some extent, conserved S1 Fig. These genes are also conserved in the genomic location where the human *RPRM* gene is located S1 Fig.

To date, only the *RPRM* paralog has been studied. *RPRM* is a highly glycosylated cytoplasmic protein, involved in p53-induced cell cycle arrest at G2 through the regulation of the Cdc2-cyclin B1 complex activity by an as yet undiscovered G2/M checkpoint mechanism [2, 3]. *RPRM* has also been suggested as a p53-dependent putative tumor suppressor gene [3]. *In vitro* studies have shown that overexpression of *RPRM* results in reduced cell proliferation, migration and invasion [4, 5], as well as increased apoptosis [6]. *RPRM* is a potential class II (inactivation occurring by epigenetic mechanisms) tumor suppressor gene (TSG). Inactivation is driven principally by aberrant methylation of its promoter region [4, 7, 8] and correlates with genetic instability in human cancers, such as gastrointestinal tumors [9, 10]. In addition, aberrant methylation of its promoter region has been proposed as a biomarker for non-invasive detection of gastric cancer [7]. Interestingly, no literature exists on the presence or role of *Reprimo-like* (*RPRML*) protein in either physiology or pathophysiology.

### Model organism

Comparative genomics have gained medical relevance, as further insights into animal genetics provide information about conservation between species and serve to develop new animal models to study human diseases [11, 12]. The zebrafish is a relevant model organism for the analysis of vertebrate development, and has become a popular organism for the study of human cancers [12]. Some advantages of this animal model include the transparency and complexity of the zebrafish embryos, combined with the easy-to-use and high-throughput features of *in vitro* models [13]. Additionally, many of the components that regulate angiogenesis and cancer are conserved between zebrafish and humans [14, 15]. Recently, we described the evolution of the *RPRM* protein family members among vertebrates and described three distinct gene lineages: *RPRM*, *RPRML* and *RPRM3* [1]. To date, no robust *in vivo* models exist to examine the temporal and spatial expression patterns of *RPRM* products during normal and pathological processes.

Herein, we characterize the temporal and spatial expression patterns of *RPRM* paralogs during vertebrate development *in vivo*, by the use of a zebrafish development model. We confirm that all three *RPRM* gene family members (*RPRM*, *RPRML* and *RPRM3*) have been retained, and are expressed in zebrafish. In the zebrafish, two copies of the *RPRM* othologue are present, termed *rprma* and *rprmb*, which represent an infraclass duplication event within the teleost lineage. In zebrafish, *rprm* (*rprma*, *rprmb)* and *rprml* are expressed during central nervous system (CNS), blood vessel and digestive tube formation. To our knowledge, these data constitute the first evidence of *RPRM* gene family expression during embryonic development, including the recently described member of the gene family, *RPRM3*. Furthermore, we show that the expression pattern of the *RPRM* orthologue is conserved between zebrafish and humans. The strong conservation in *RPRM* gene expression patterns in various tissues between fish and human, suggest that zebrafish may serve as a useful model organism to identify and study the function of *RPRM* genes in organismal development, as well as physiological and pathological processes.

## Materials and methods

### Multiple sequence alignment

In order to identify potential differentiating amino acid substitutions to distinguish between *RPRM*, *RPRML* and *RPRM3*, we annotated *RPRM* genes in human, mouse and zebrafish. Amino acid sequences were aligned using the L-INS-i strategy from MAFFT v.7 [16]. Additionally, potential domains were predicted using the TMHMM method (http://www.cbs.dtu.dk/cgi-bin/nph-sw_request?tmhmm), as implemented in Geneius Software.

### RNA extraction and reverse-transcription of cDNA templates from zebrafish larvae

For developmental expression analysis of *rprma*, *rprmb*, *rprml* and *rprm3*; embryos were collected after timed intervals of 0.2, 6, 12, 24, 31, 48, 72 and 96 hours post-fertilization (hpf), quick-frozen on liquid nitrogen, and stored at -70°C until analysis (3 independent embryo pools, 50 embryos per pool, per time point from the same spawning group). Whole embryos were homogenized in Lysis Buffer (Thermo Scientific) and total RNA was extracted with GeneJet RNA Purification kit (Thermo Scientific) according to manufacturer's instructions. Embryo *actin*, *beta 1* (*actb1*) was used for housekeeping gene expression analysis and gene of interest normalization. An aliquot (2μl) of each extract was used for RNA quantification, quality assessment and concentration by spectrophotometry (BioSpectrometer, (Eppendorf)). RNA with a 260/280 ratio between 1.6–2.0 was considered optimal in this study. Each RNA extract was performed in triplicate and an average value was determined. cDNA was synthesized from total RNA (1 μg; 20 μl final reaction volume) with oligo (dT) priming using ImProm-II Reverse transcriptase II (PROMEGA) according to manufacturer's instructions.

### Dissection, RNA extraction and reverse-transcription of RNA templates from adult zebrafish tissues

For adult expression analysis of *rprma*, *rprmb* and *rprml*, 4 adult TAB5 wild-type male zebrafish were euthanized for organ extraction, according to the protocol by Gupta & Mullins (10.3791/1717). Zebrafish brain and bowel were harvested and homogenized in TRI-Reagent^®^ (Sigma-Aldrich). Total RNA was collected separately for each sample, according to manufacturer’s instructions. A 2μl aliquot of each extract was used for RNA quantification, quality assessment and concentration by spectrophotometry (BioSpectrometer, (Eppendorf)).

cDNA was synthesized from total RNA (1 μg; 20 μl final reaction volume) with oligo (dT) priming using ImProm-II Reverse transcriptase II (PROMEGA) according to manufacturer's instructions.

### Reverse-transcription quantitative polymerase chain reaction (RT-qPCR)

All real-time PCR experiments were performed using a Stratagene Mx3000P detector system (Agilent Technologies) with optic tubes (SSI Innovations for Life Science). Amplification was achieved using Brilliant II SYBR^®^ Green QPCR Master Mix (Agilent Technologies), equal amount of cDNA, and gene specific primers sets: *rprma*, fw: 5- AACCAAACGGACAGTGGCATCT-3’, rv: 5’- AAGACTACGGTGAGGGAAAGCA-3’; *rprmb*, fw: 5’- GGCTGCAACTTGCTGATTAAGTCC-3’, rv: 5’- GATGACCGCTTCCACATCCTTTGA-3’; *rprml*, fw: 5- ACGAGCGCAAACTGTTCGTTAC-3’, rv: 5- TCA TGAGGTTGCAGCCGAGAA A-3’; *rprm3*, fw: 5’- TGTTTCTCACGGACTACTGAACC-3’, rv: 5’- TAATACGACTCACTATAGGGTGCAGCGATCATAATAATTTCC-3’. Following completion of each real-time PCR reaction, a dissociation step was added and melt curve analysis was performed to validate the specificity of PCR amplicons. Data were processed by MxPro qPCR Software 4.10. *rprm* mRNA expression levels were normalized against an endogenous control gene previously validated as suitable reference gene for developmental studies in zebrafish [17]: *actb1*: fw:5’ -CGAGCAGGAGATGGGAACC-3’, rv: 5’-CAACGGAAACGCTCATTGC- 3’. Relative expression was calculated by ΔCT method against the housekeeping gene *actb1*. Expression plots were elaborated using R statistical programming language package ggplot2 and represented as average relative expression +/- SEM or relative expression boxplots, for developmental and adult expression, respectively.

### Zebrafish husbandry, whole-mount in situ hybridization (WISH) and sections

Wild-type (TAB5) and transgenic reporter zebrafish (*Danio Rerio*) were maintained according to standard methods [18]. Embryos were raised in system water at 28°C and staged according to either hours post-fertilization (hpf) or morphological criteria [19]. WISH was carried out as described previously [20]. For WISH experiments, embryos older than 24hpf were treated with 0.003% 1-phenyl 2-thiourea (Sigma) to inhibit pigmentation. To better characterize the expression domains of the *rprm* genes, embryos stained with riboprobes were incubated in 30% sacarose/PBS/azide and then embedded in OCT (optimal cutting temperature) compound (Sakura). The embryos were sectioned by microtome-cryostat (Leica CM 1510S) in a sets of serial transverse/coronal sections (25 mm of thickness). The sections were collected in super frost covers then dehydrated and mounted. The pictures were captured with DS-U3 Nikon camera, using the SMZ18 stereomicroscope, Nikon. All the zebrafish studies were conducted under the guidance and approval of the Institutional Animal Care and Bioethical Committee at Pontificia Universidad Católica de Chile.

### cRNA probe synthesis for WISH

Templates for probe synthesis were PCR amplified from embryonic zebrafish cDNA using primers including T7 RNA polymerase promoter sequence. To minimize cross-reactivity, the 5’ untranslated (5’-UTR) regions of *rprm* genes were used for primer design. Primer sets were designed as follows: *rprma*, fw: 5’- TGAGGAGAACCTCCTGTGCT-3’, rv: 5’-TAA TACGACTCACTATAGGGGCCTGATCCTGATGGTTCGT-3’; *rprmb*, fw: 5’- TCCACCCATTCATCCTGTCA-3’, rv: 5’- TAATACGACTCACTATAGGGTCGGAGTTTCTTCGTTTGTG-3’; *rprml*, fw: 5-GACCGGAGATCATCCAAAGA-3’, rv: 5’-TAATACGACTCACTATAGGGCTCG TTTCGTAAACGTGCAA-3’; and *rprm3*, fw: 5’- TGTTTCTCACGGACTACTGAACC-3’, rv: 5’- TAATACGACTCACTATAGGGTGCAGCGATCATAATAATTTCC-3’. All PCR products were of the expected size as inspected by agarose gel electrophoresis. Purified PCR products were *in vitro* transcribed and labeled using digoxigenin (DIG) RNA labeling Kit (Roche) according to manufacturer’s protocol. cRNA probes were purified by mini Quick Spin RNA Columns (Roche) and stored at -80°C with deionized formamide.

### Fluorescent whole-mount in situ hybridization (FISH)

Embryos were fixed in 4% paraformaldehyde (PFA) in PBS at specified times post-fertilization. Samples were permeabilized with 50 μg/ml Proteinase K for 30 minutes at room temperature. Samples were then prehybridized in HYB+ with 0.1% tween SSCT at 68°C followed by incubation with antisense probes against *rprm* or *pdgfrβ* that were labeled with either Dig—or Fluorescein RNA labeling MIX (Roche) over night. Probes were detected with anti-DIG or anti Fluorescein antibody (1:500) conjugated horseradish peroxidase (Roche). Signal was produced by treatment with TSA plus Cyanine 3/Fluorescein system (PerkinElmer). Immunofluorescence was completed using rabbit anti-GFP (1:500, Life technologies), and primary antibodies were detected using anti-rabbit antibody conjugated to Alexa-488/Alexa-647 (1:500, invitrogen).

### Imaging

Zebrafish embryos for WISH were embedded in 75% glycerol/PBS and imaged using NIKON eclipse 80i microscope equipped with a DS-Vi1 (NIKON) camera. Fluorescent images of embryos were acquired using Zeiss LSM780 laser-scanning confocal microscope.

### Immunohistochemistry staining

Immunohistochemistry for *RPRM* (Anti-*RPRM* (38–50) antibody, Sigma-Aldrich) was performed on 4μm whole sections containing human stomach, both antral and fundic-corporal mucosa, human brain cortex, including white and gray matter, and human small bowel, obtained from the archives of the Pathology Department, Hospital Clínico Universidad de Chile, by Vectastain Elite Kit R.T.U (Vector Labs), according to manufacturer’s instructions, as previously described [3]. Evaluation of immunohistochemical staining was performed independently by a pathologist (GC) who was blinded to ongoing lab results.

## Results

### Protein alignments

**Fig 2:**
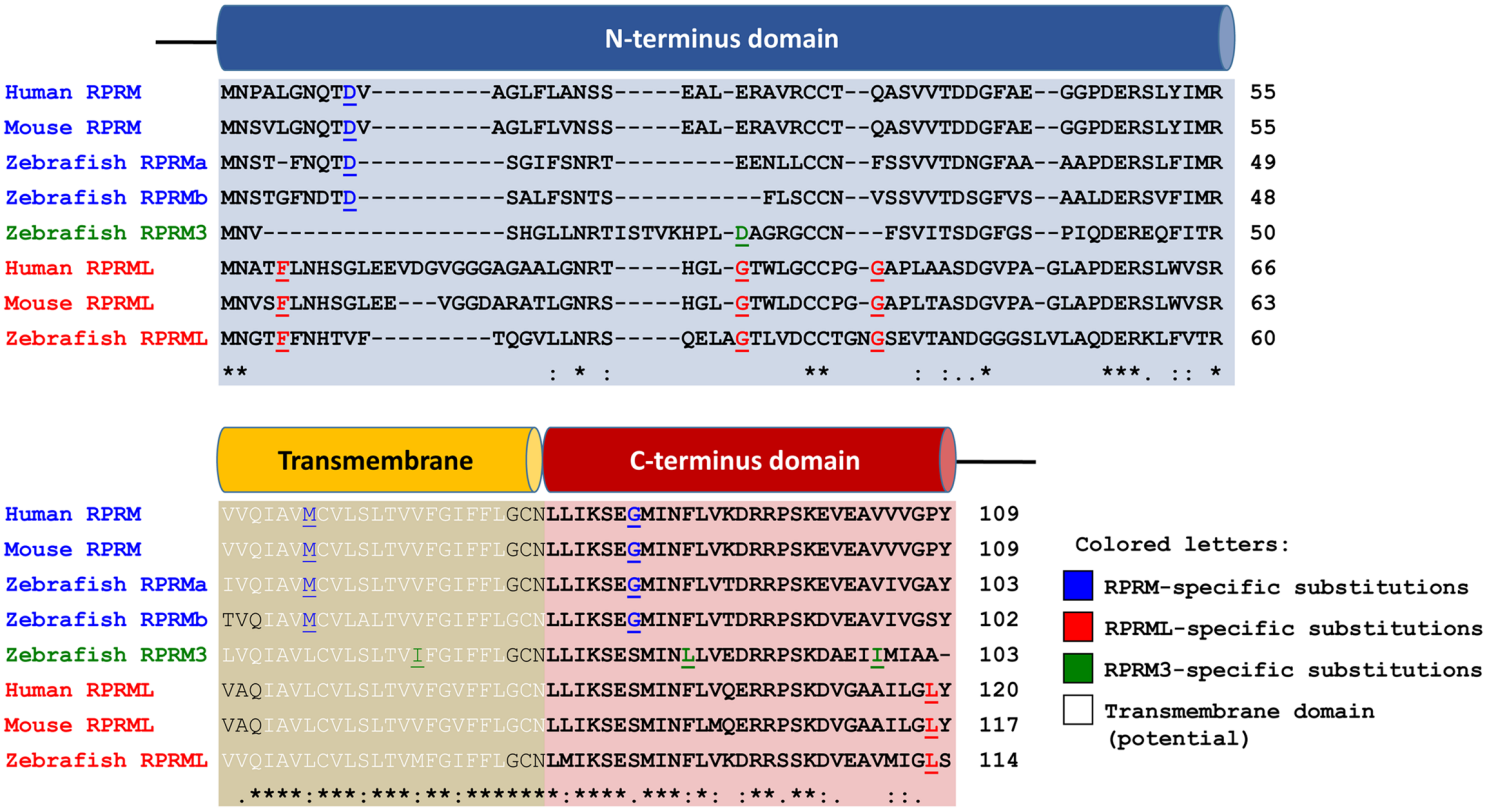
*RPRM* protein family multiple sequence alignment. The N-terminus of the proteins shows most of the sequence variance and insertions, while the C-terminus is greatly conserved. Some amino acid substitutions can be observed which mark differences between the two proteins. *RPRM*- and *RPRML*-specific amino acids are highlighted in blue and red, respectively. An * (asterisk) indicates positions which have a single, fully conserved residue. A: (colon) indicates conservation between groups of strongly similar properties—scoring > 0.5 in the Gonnet PAM 250 matrix. A. (period) indicates conservation between groups of weakly similar properties—scoring ≤ 0.5 in the Gonnet PAM 250 matrix. Potential transmembrane domain predicted using Geneius software.

Multiple sequence alignment of *RPRM*, *RPRML* and *RPRM3* proteins between human, mouse and zebrafish showed a striking conservation of the C-terminus across species (Fig 2), while the N-terminus domain retained the most variability within the protein family. Analysis of the protein sequence by TMHMM method, revealed a short region of ~20 amino acids conforming a potential transmembrane domain in the three proteins. Amino acid differences were identified between the protein sequences, which may serve to distinguish each lineage from the others (Fig 2), colored letters in alignment; S1 Table). Aspartic acid (D), methionine (M) and glycine (G) at conserved sites serve to distinguish *RPRM* from *RPRML* and *RPRM3*. Phenylalanine (F), glycine (G) and leucine (L) at conserved sites serve to distinguish *RPRML* from *RPRM* and *RPRM3*. Aspartic acid (D), glycine (G), isoleucine (I) and leucine (L) at conserved sites serve to distinguish *RPRM3* from *RPRM* and *RPRML*. The high level of conservation (72.5% and 66% identity between human *RPRM* and zebrafish *rprma*, *rprmb* and *rprm3* proteins, respectively; 55.7% identity between human and zebrafish *RPRML* proteins) observed in the *RPRM* protein sequences and domains suggests the functions of the proteins may be related; though further research is warranted to support this conclusion.

### Developmental expression profiles of *RPRM-*paralogs in zebrafish

Gene expression profiles of *rprm* (*rprma* and *rprmb)*, *rprml* and *rprm3* paralogs were determined using RT-qPCR on RNA extracted from whole zebrafish embryos and larvae, ranging from 0.75 to 96 hours post-fertilization (hpf) S2 Fig. In zebrafish, all *rprm* transcripts were expressed at low levels. At 12 hpf, *rprmb* transcripts were the most abundantly expressed, being expressed more than 3 times relative to *rprma*, *rprml* and *rprm3* S2 Fig. All *rprm* genes shared a similar RT-qPCR expression profile, with highest levels for *rprmb* at 12 hpf and an ascending trend, for all them (*rprma*, *b* and *l*), towards 4 days post-fertilization (dpf) S3 Fig.

**Fig 3:**
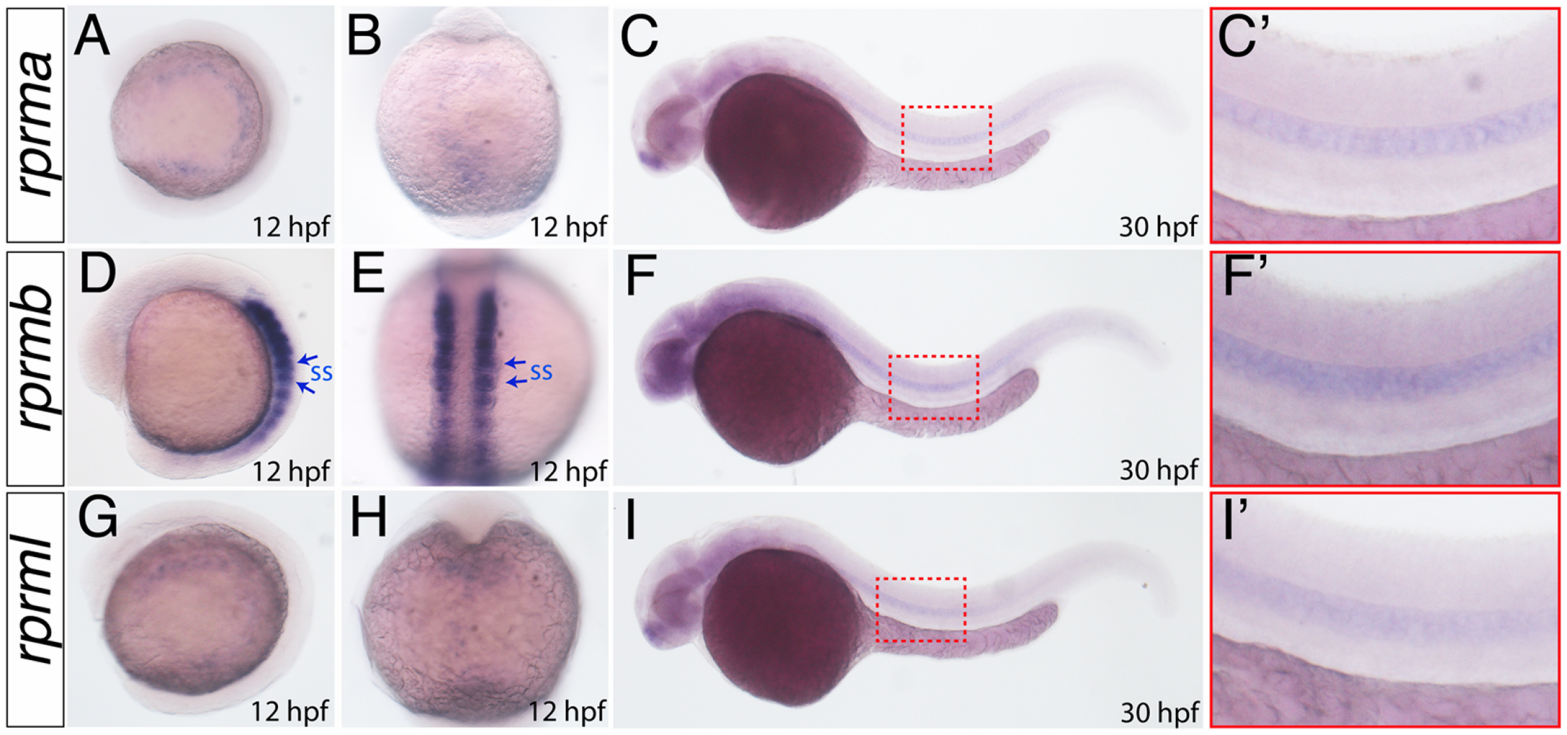
*rprm* expression patterns during early embryogenesis. (A-I) The expression patterns of *rprma*, *rprmb* and *rprml* were visualized by whole-mount in situ hybridization (WISH) during zebrafish embryonic development. Developmental stages are expressed as hours post-fertilization (hpf). (A, C, D, F, G, I) Lateral (anterior to the left) and (B, E, H) dorsal views are shown. Blue arrows in D-E indicate the expression in the somites (ss), while (C, F, I) the red inset magnifications indicate the expression in the notochord.

To compare the spatio-temporal expression of the *rprm* transcripts, zebrafish of various developmental stages were subjected to WISH using gene-specific complementary RNA probes. Consistent with the RT-qPCR data, At 12 hpf, *rprmb* was detected at higher levels in the somites (ss) territories during early somitogenesis (5–12 ss) (Fig 3D and 3E), while *rprma and rprml* lacked staining on ss (Fig 3A and 3B), (Fig 3G and 3H). At 24 hpf, the three *rprm* genes were expressed in the notochord (Fig 3C), (Fig 3F and 3I). Weaker and more diffuse staining was also detected in the lateral portion of paraxial mesoderm (data not shown).

### *RPRM* genes are expressed in zebrafish and human brain

**Fig 4:**
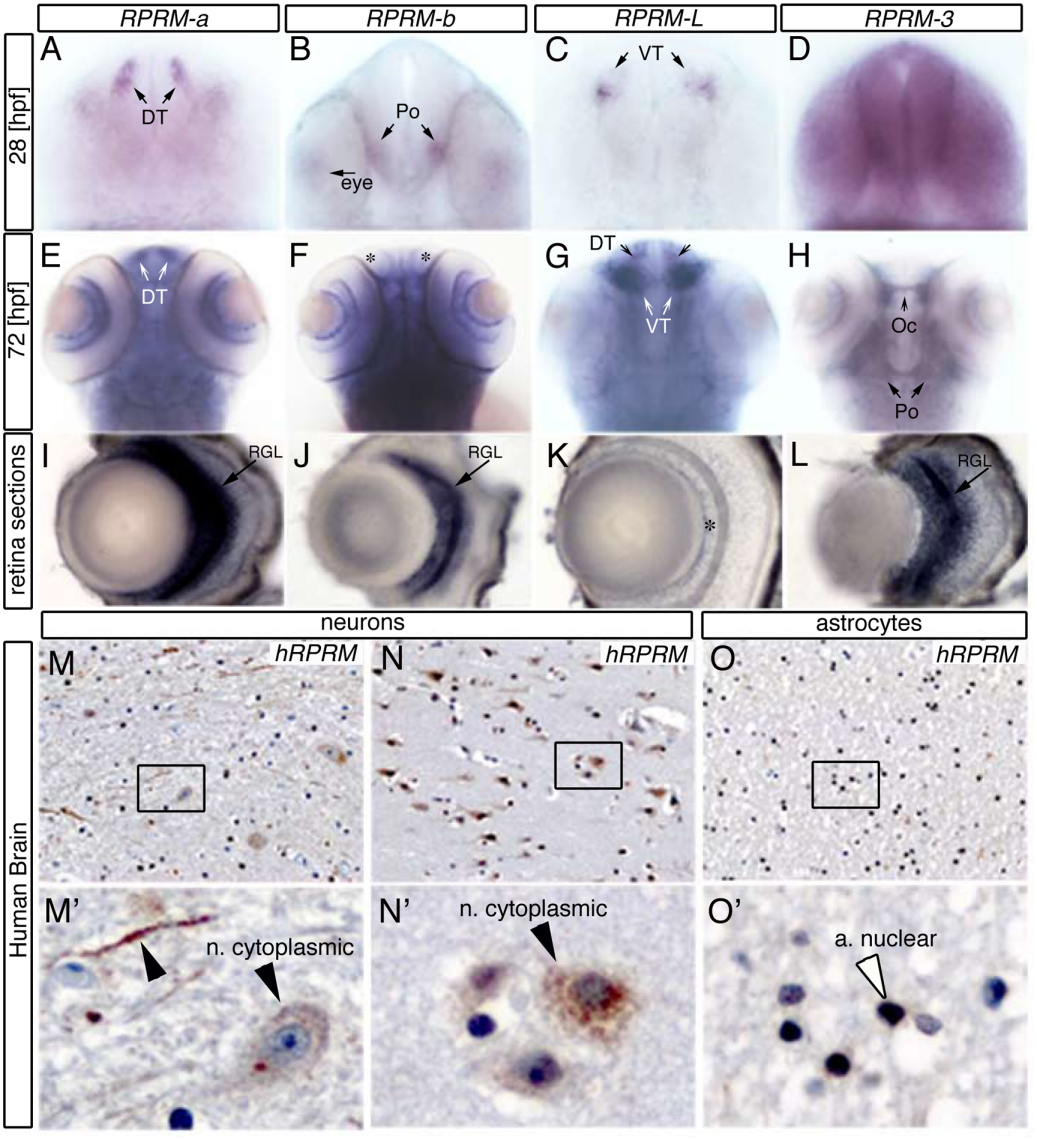
*RPRM* expression patterns are conserved between zebrafish and human brain. (A-D) *RPRM* expression patterns were examined using whole-mount in situ hybridization in wild-type embryos at 28 and (E-H) 72 hours post-fertilization [hpf]. (A-D) Frontal views of the embryo heads. (E-H) Dorsal views of the embryo heads. (I-L) Retina cross-sections. (A-C) At 28 hpf *rprma*, *rprmb* and *rprml* transcripts are expressed in neuronal populations such as dorsal thalamus (DT), preoptic region (Po) and ventral thalamus, respectively (black arrows). (D) *rprm3* is ubiquitously expressed throughout the brain. (E-G) At 72hpf *rprma* and *rprml* are expressed in the DT and the VT, respectively (white arrows), while *rprmb* is not expressed in those regions (asterisks). (H) At the same developmental stage, *rprm3* mRNA is expressed in the Po and the optic chiasma (Oc). (I-L) Cross sections of the retina. (I, J, L) *rprma*, *rprmb* and *rprm3* expression are restricted in the retina to the retinal ganglion cell layer (RGL, black arrow). (K) In contrast, *rprml* transcript expression is absent in the RGL (asterisk). (M, M’, N, N’) IHC staining for *RPRM* of white and grey matter sections from adult human samples (400x; inset magnifications 600x). (M-N) *RPRM* protein is expressed in the cytoplasm and axons of neurons (black arrowheads). (O-O’) *RPRM* protein is expressed in the nuclei of astrocytes.

For all four *rprm* transcripts, we were able to detect restricted and specific hybridization in the brain and the CNS around one-day post-fertilization (Fig 4A–4D) and S4 Fig. At this developmental stage, transcript expression of *rprma* and *rprml* was observed in the forebrain (fb) in two bilateral cell populations at the dorsal (DT) and ventral thalamus (VT), respectively (Fig 4A and 4C). *rprmb* expression was evident in two discrete domains within the preoptic region (Po) (Fig 4B) and the cranial placode in the trigeminal ganglia S4 Fig.

By 72hpf, *rprma*, *rprmb* and *rprml* maintained their expression in the fb territories (Fig 4E–4G), while *rprm3* staining was apparent in discrete regions of the midbrain (mb) (Fig 4H). We confirmed the expression of *rprma* and *rprml* in the telencephalic neurons performing whole-mount fluorescent *in situ* hybridization (FISH) (S5 Fig). In the retina, *rprma*, *rprmb* and *rprm3* positive cells were clearly expressed in the retinal ganglion cell layer (RGL) (Fig 4I), (Fig 4J and 4L). *rprml* was not expressed in the retina (Fig 4K). These results indicate that *rprm* (*rprma/rprmb*)*/rpml* gene expression partially overlap during zebrafish brain development. Interestingly, *rprm3*, which has been differentially retained only by some fish, showed the most unique pattern of expression in comparison to *rprm* (*rprma/rprmb*)*/rprml* genes during the development of the brain/CNS.

Adult zebrafish relative expression of *RPRM* transcripts within the brain was evaluated by RT-qPCR, with the exception of *rprm3* which was not assessed due the lack of this gene in humans (S6 Fig, S2 Table for raw RT-qPCR data). *rprml* showed the highest expression, followed by *rprma*. Interestingly, *rprmb* showed very low expression levels in the adult zebrafish brain S6 Fig.

Human *RPRM* protein expression was examined by immunohistochemistry (IHC) staining, using rabbit polyclonal anti-*RPRM* antibody. In the adult human brain cortex, *RPRM* protein was localized at the cytoplasm of the body and axons of neurons (Fig 4M and 4N); and at the nuclei of astrocytes (Fig 4O).

### *RPRM* genes are expressed in zebrafish and human vascular tissues

The expression of *rprm* genes in vascular tissues begins around 48hpf. In the head, *rprm*-positive cells coat blood vessels such as central artery (CtA), primordial hindbrain channel (PHBC), mesencephalic vein (MsV), dorsal longitudinal vein (DLV), primary head sinus (PHS), nasal ciliary artery (NCA) and inner optic circle (IOC) S7 Fig. In addition, *rprm*-positive cells clustered together in the hypochord (Hp) posterior cardinal vein (PCV) and sprouting intersegmental vessels (ISV) S7 Fig. *rprm*-positive cells were scattered throughout the brain, consistent with the possibility that *rprm* expression marks mural cells associated with the blood vessels S7 Fig (inset magnifications). To determine which cell types expressed *rprm* genes, we performed FISH followed by confocal microscopy. To visualize vasculature, we used the transgenic reporter *Tg (kdrl*:*EGFP)*^*s843*^ and *pdgfrβ*, which label vascular endothelial and mural cells, respectively S8 Fig. In these experiments, we did not observe detectable co-localization. This was likely because the *rprm* genes are expressed at very low levels S2 Fig. It is important to note that, despite the use of signal amplification systems for increasing sensibility, FISH detection in whole-mounts suffers for relatively low signal sensibility and signal-to-noise ratio. Thus, weakly expressed genes such a *rprms* cannot be efficiently detected.

**Fig 5:**
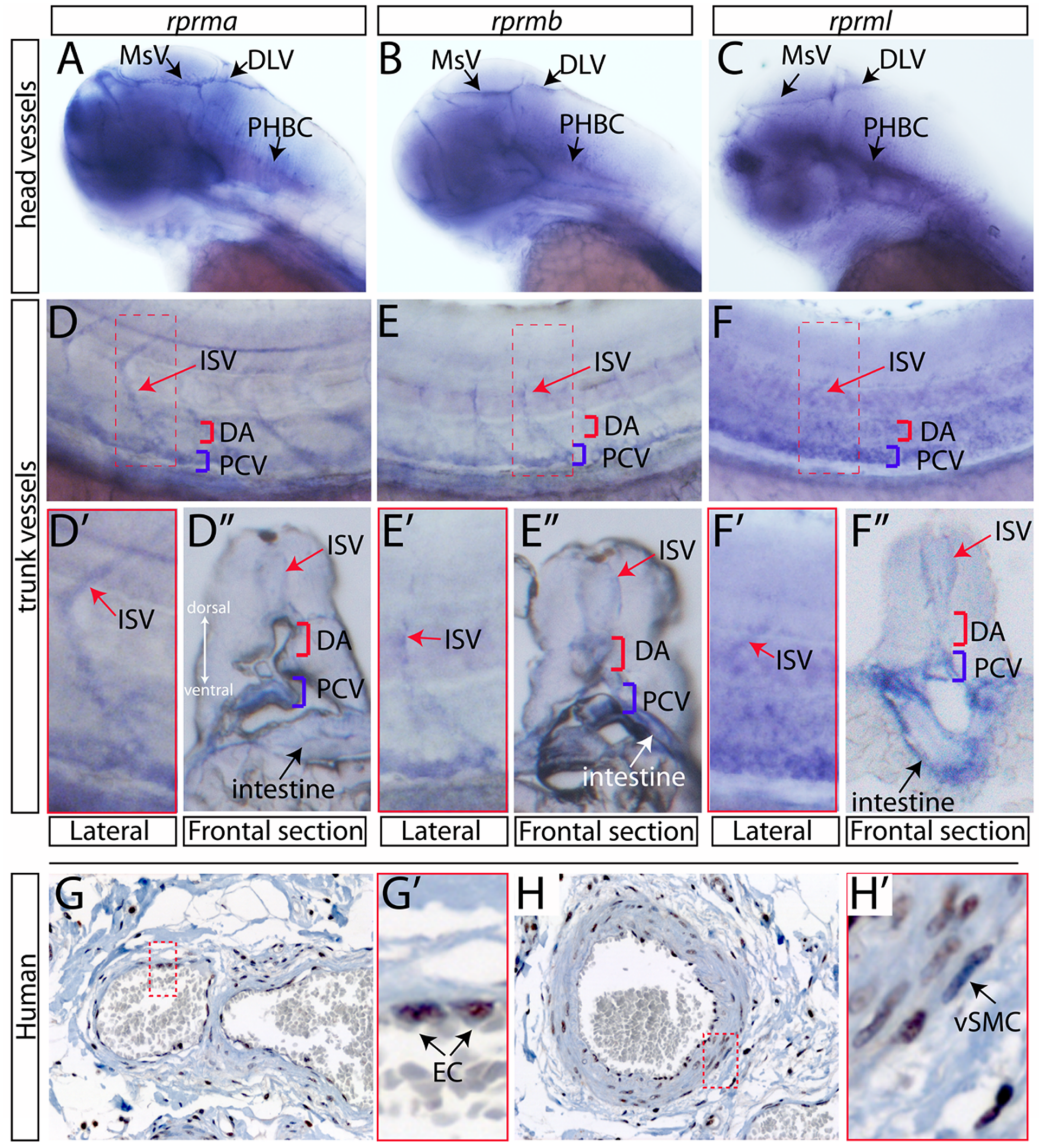
*RPRM* is expressed during zebrafish angiogenesis and in adult human vasculature. (A-C). Whole-mount in situ hybridization at 72hpf. Lateral view of the head vasculature. *rprma/b* and *l* are expressed in the mesencephalic vein (MsV), the dorsal longitudinal vein (DLV) and the primordial hindbrain channel (PHBC). (D-F) Lateral view of the trunk vessels. *rprma/b* and *l* are expressed at low levels in the dorsal aorta (DA, red bracket) the posterior cardinal vein (PCV, blue bracket) and the intersegmental vessels (ISV, red arrow). (D’-F’) Inset magnifications (red brackets) and (D”-F”) transverse histological cross-sections of the posterior trunk region. (G-H) IHC staining for *RPRM* in tissue sections showing small blood vessels from adult human samples (400x; inset magnification 600x). (G, G’) *RPRM* protein is expressed in the nuclei of endothelial cells (EC, black arrows) from a small artery. (H-H’) *RPRM* protein is expressed in the nuclei of EC and vascular smooth muscle cells (vSMCs, black arrow) of a muscular artery.

At 72 hpf, the embryos maintained the expression of *rprma*, *rprmb* and *rprml* in brain blood vessels such as MsV, DLV and PHBC (Fig 5A and 5C). In the trunk vessels, *rprma rprmb* and *rprml* transcripts were expressed at low levels in the dorsal aorta (DA), the PCV and the ISV (Fig 5D–5F”). Interestingly, we did not detect vascular expression of *rprm3* during zebrafish blood vessel development (data not shown). Importantly, cells positive for *rprm* expression in vascular endothelial cells were not detected before 48 hpf (Fig 3), which indicate that *rprm* might participate in later stages of vascular commitment in the zebrafish embryos.

*RPRM* IHC in human samples revealed nuclear *RPRM* protein expression in endothelial cells of medium size arteries and capillaries within the wall of the digestive tube (i.e. small bowel) (Fig 5G and 5G’), as well as in the nuclei of vascular smooth muscle cells (vSMCs, also known as mural cells) of muscular arteries within the small intestine wall (Fig 5H and 5H’). The distribution observed with *RPRM* antibody was consistent with *rprm* mRNA expression during zebrafish angiogenesis.

### *RPRM* genes are expressed in zebrafish and human digestive tract

**Fig 6:**
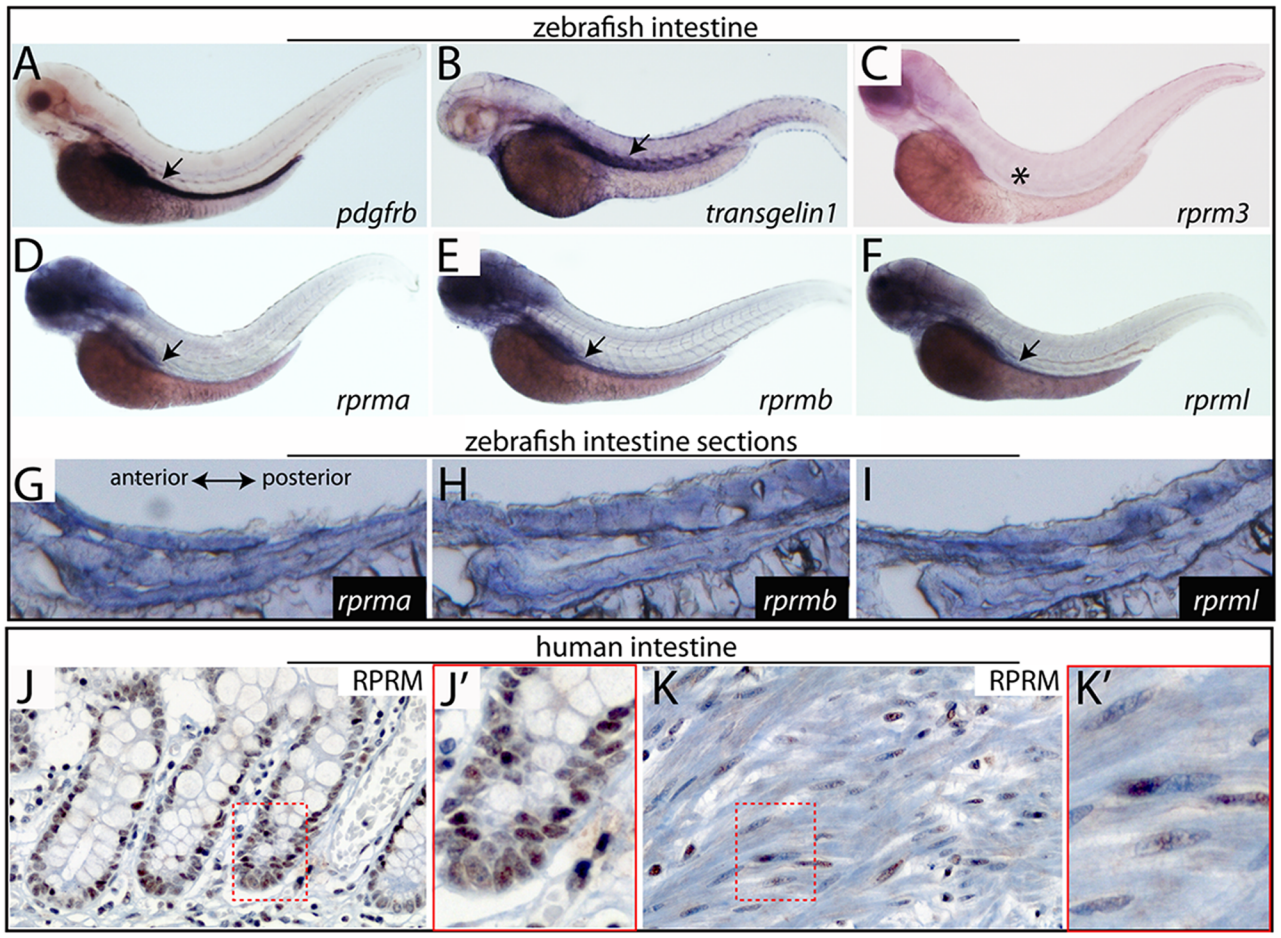
Expression of *rprm* genes during zebrafish gut development and human intestinal tissue. (A-F) Lateral views of whole-mount in situ hybridization (WISH) at 72hpf. (A-B) WISH for two well-known gut markers (A) platelet-derived growth factor receptor (*pdgfrβ*), (B) smooth muscle actin (*sm22*, also known as *transgelin1*), and the *rprm* genes (C) *rprm3*, (D) *rprma* (E) *rprmb* and (F) *rprml*. At 3dpf, *rprma/-b* and *rprml* are expressed in the embryonic intestinal tube. At the same developmental stage, there is a lack of expression of *rprm3* in the intestinal tissue (C, asterisk). (G-I) Lateral cross sections of the zebrafish gut, anterior to the left. (J-J’) *RPRM* IHC staining (400x; inset magnification 600x) showing nuclear positive expression in enterocytes at the base of the intestinal crypts. (K-K’) *RPRM* IHC, (400x; inset magnification 600x) showing nuclear positive expression in smooth muscle cells from the muscularis propria of the small intestine.

WISH was performed to examine *rprm* gene expression in developing digestive tissues. As markers for intestinal territory, we used platelet derived growth factor beta (*pdgfrβ*) and smooth muscle alpha actin (*sm22*, also known as *transgelin1*) (Fig 6A and 6B). As the digestive organs developed, the three *rprm* (*rprma*, *rprmb* and *rprml*) genes were expressed throughout the length of the intestine. This expression pattern became increasingly prominent between 72 to 96 hpf (Fig 6D and 6I). This time period corresponds to the maturation of intestinal epithelium [21]. Of note, *rprm3* was not expressed on endoderm-derived intestinal tissues (Fig 6C). We did not detect *rprm* genes in other endoderm-derived organs such as the liver, in any stages studied.

Adult zebrafish relative expression levels of *rprm* transcripts within the intestine was evaluated by RT-qPCR S6 Fig, with the exception of the *rprm3* transcript which was not expressed in this tissue, as assessed by WISH in zebrafish embryos and due to the lack of this gene in humans. Transcripts for *rprma* and *rprml* show the highest expression levels, whereas relative expression of *rprmb* was practically undetectable S6 Fig.

*RPRM* IHC in human small bowel and stomach samples revealed expression of *RPRM* protein in the nuclei of normal enterocytes from the small bowel, especially at the base of the crypts (Fig 6J and 6J’), and in the nuclei of smooth muscle cells of the muscularis propia (Fig 6K and 6K’). *RPRM* IHC was also positive in the nuclei of normal gastric foveolar epithelium and in the nuclei of normal gastric antral and fundic glands. When compared with a recently reported work from Saavedra et al [4], in which *RPRM* expression was observed in the cytoplasm of gastric cancer non-tumor adjacent mucosa (NTAM), only a faint positivity was observed in the cytoplasm of foveolar and antral/fundic glands epithelium.

## Discussion

It has been shown that *RPRM* plays a critical role during carcinogenesis [4, 22–26]. Nevertheless, the expression patterns and developmental transcript localization of *RPRM* has not been previously reported. Moreover, no studies have reported the transcript localization or functions of *RPRML* or *RPRM3*. In this study, we investigated for the first time the transcriptional expression profiles of *rprm* genes during zebrafish development. We used WISH to demonstrate that all of the *rprm* genes (*rprm (rprma/rprmb)*, *rprml* and *rprm3*) were expressed during normal zebrafish embryogenesis. Gene expression profile analysis of the *rprm* genes demonstrated that they exhibit unique, although partially overlapping expression patterns during embryonic and larval development; in the brain, gut and vasculature. Most importantly, the expression patterns of *rprm* (*rprma* and *rprmb)* transcripts in zebrafish resembled human expression profiles of the *RPRM* protein product, as demonstrated by IHC staining. Note that *rprmb* is expressed primarily during developmental phases in zebrafish larvae, but expression is lost in adult zebrafish. This suggests that this duplicated gene most probably plays an important role that is restricted to development. These findings indicate that *RPRM* expression is conserved in fish and humans, subsequently validating the use of this model organism for the functional characterization of *RPRM* in development, biology and disease.

*RPRM* genes appear to be an evolutionary innovation of vertebrates as no traces of them have been found in urochordates. This result is in agreement with reports that suggest that a relatively high proportion of disease related genes originated in the last common ancestor of vertebrates [27]. *RPRM* genes expanded as a consequence of the two rounds of whole genome duplications that occurred early in the evolutionary history of vertebrates [28–32]. Although four gene copies are expected as a result of this process only three *RPRM* genes have been described in vertebrates to date (Fig 1; *RPRM*, *RPRML* and *RPRM3*;[1]), suggesting that one *RPRM* copy was lost shortly after a whole genome duplication event. During the evolutionary history of teleost fish (e.g. zebrafish), a third round of whole genome duplication occurred in the common ancestor of the group [33–35] that further expanded the repertoire of *RPRM* genes. The resultant *RPRM* orthologues have been differentially retained during the evolutionary history of teleosts. In the case of *RPRM*, both orthologues derived from the teleost-specific genome duplication are present in the zebrafish (*Danio rerio*) and cave fish (*Astyanax mexicanus*). For the other two *Reprimo* genes (*RPRML* and *RPRM3*), only one of the lineages has been retained. The most interesting case is the restricted phyletic distribution of *RPRM3* that among teleost fish was only identified in zebrafish and cavefish.

At the amino acid level, there is a striking conservation throughout the C-terminus of the *RPRM* family, suggesting a strong selective pressure on the composition of this domain (Fig 2). The conservation of the protein sequences between zebrafish and human *RPRM* proteins, as well as the conservation of the expression pattern of these genes, suggest that *RPRM* proteins might play homologous roles in zebrafish and humans. Therefore, zebrafish could be proposed as a model organism for the functional characterization of *RPRM* proteins.

RT-qPCR revealed the expression of *rprm* genes during embryonic development in zebrafish. *rprm* (*rprma* and *rprmb*) and *rprml* genes were detected in the forebrain and telencephalon at early developmental stages, while *rprm3* expression became progressively restricted to the midbrain (Fig 4). These genes were also detected in developing retina where they had partially overlapping expression.

In human brain, IHC staining revealed that *RPRM* was expressed in the neuronal cytoplasm and astrocyte nuclei (Fig 4). Together, the high degree of evolutionary conservation for *RPRM/RPRML* and the brain-specific expression observed between zebrafish and humans indicate that *RPRM* might be essential for brain development and/or function. Future studies using *RPRM* knockdown models will be required to determine if *RPRM* genotypic expression gives rise to a phenotype.

As in fish, human *RPRM* was expressed in the vasculature (Fig 5); specifically, in vascular smooth muscle and endothelial cells. In zebrafish, after 2dpf, the *sm22* and *acta2* label mural cells in perivascular tissues [36]. The appearance of the first *RPRM*(+)-cells in vascular tissues was observed after 2dpf (Fig 5) and S7 Fig. However, we did not observe significant fluorescent co-localization between *RPRM*(+)-cells and mural cells markers S8 Fig. Of note, fluorescent-labeled probes (FISH) are generally weaker than digoxygenin-labeled probes (WISH) for the same gene. Therefore, determining the expression pattern for particularly low-abundance transcripts such as *RPRMs* S2 Fig might not be feasible by FISH in perivascular tissues.

*rprm* and *rprml* expression were detected in zebrafish intestine. *RPRM* protein expression was also detected in the human gastrointestinal tract. In the latter, we observed expression of *RPRM* protein in regions of the muscularis propia surrounding the intestine (Fig 6). Histological analyses show that the zebrafish intestine is arranged in concentric smooth muscle cell layers similar to those present within the mammalian intestine [21]. This finding was consistent with previous reports indicating that the zebrafish smooth muscle layers are in correspondence with the mammalian muscularis propia [21]. In human tissue, *RPRM* protein expression was observed in the nuclei of small bowel epithelial cells, especially at the base of the crypts. *RPRM* expression was also observed in the nuclei of human normal gastric epithelia; at foveolar epithelium and glands from the antrum and fundic regions. Interestingly, when compared with previous work of Saavedra et al [4], *RPRM* was mainly expressed in the nuclei, rather the cytoplasm, of foveolar and gastric glands epithelium, showing a faint positivity in the cytoplasm. The main difference with Saavedra et al., is that cytoplasmic *RPRM* positivity was found in gastric cancer NTAM, a mucosa with atrophy and intestinal metaplasia, both known to be premalignant lesions of the precancerous cascade of gastric cancer [37], but our case represent normal gastric human adult tissue. We speculate that *RPRM* expression and cellular location of *RPRM* may change during the precancerous cascade of gastric cancer, opening the door to future research studies.

In summary, we have isolated the *RPRM* vertebrate genes and have characterized their genetic map position, evolution and expression patterns in zebrafish and humans. Our results identify potential structural domains in *RPRM* and *RPRML* subfamilies and describe their probable evolutionary history. Taken together, the distinctive spatiotemporal localizations of the *RPRM* gene expression patterns in zebrafish suggest that these paralogs have undergone subfunctionalization through evolution. Furthermore, our evidence suggests that developmental function for the *RPRM* gene family in blood vessel formation has likely been conserved in fish and mammals. Expression during CNS formation is seen in fish and human genes throughout the *RPRM* family, suggesting a potential function in brain development which is likely ancestral to all *RPRM* genes. The developmental and cancer roles of these genes can now be tested in the zebrafish using overexpression and loss of function approaches. Therefore, zebrafish presents a unique opportunity to dissect the role of *RPRM* genes in specific cell types. Future efforts will utilize directed genomic inactivation using genome-editing technologies to better characterize this fascinating protein family.

## Supporting information

S1 Fig Patterns of conserved synteny in the genomic regions that harbor *RPRM* genes in humans and *rprma* and *rprmb* of zebrafish. (PDF)

S2 Fig RT-qPCR relative expression profiles of *rprm (rprma/rprmb)*, *rprml* and *rprm3* transcripts during zebrafish development. Relative mRNA expression of *rprma*, *rprmb* and *rprml* are plotted across indicated developmental points. Each point represents the average measurement of three biological replicates. Bars correspond to standard error of the mean (SEM). Hpf: hours post-fertilization. Relative expression was normalized against *actin*, *beta 1* (*actb1*).
(PDF)

S3 Fig Relative expression profile of *rprm* genes in embryonic zebrafish. Relative mRNA expression of *rprm* genes are plotted across indicated developmental points. Each point represents the average measurement of the relative expression of the 4 *rprm* genes (*rprma/rprmb*, *rprml* and *rprm3*). The dark grey area around the curve represents SEM.
(PDF)

S4 Fig *rprm* expression during brain development in zebrafish embryos. *rprm* expression patterns were examined using whole-mount in situ hybridization in wild-type embryos at (A-D) 1 day post-fertilization [hpf]. (A-D) Lateral views. (A-C) At these developmental stages, *rprma*, *rprmb* and *rprml* transcripts are located in neuronal populations such as dorsal thalamus (DT), ventral thalamus (VT) and the cranial placode in the tigreminal ganglia (tg). (D) *rprm3* is ubiquitously expressed through the brain.
(PDF)

S5 Fig *rprma* and *rprml* are expressed in brain neurons in zebrafish larvae. (A-D) *rprma* and *rprml* expression was detected by whole-mount fluorescent in situ hybridization (FISH) at 72hours post-fertilization [hpf]. (A) Confocal cropping shows *rprma* expression in the anterior neurons of the telencephalon. (C-D) Confocal sectioning shows *rprml* expression in the posterior neurons within the forebrain (inset magnification). (A’D’) Transgenic *Tg(fli1a*:*EGFP)* is expressed in the endothelial cells within the major blood vessels of the head.
(PDF)

S6 Fig Relative expression of *rprm* genes in adult zebrafish brain and intestine. *rprm* genes are expressed in the brain (red dots). *rprml* and *rprma* are the highest expressed genes in the brain (red dots) and intestine (blue dots), respectively. *rprmb* has not significant expression in either tissue. *rprm3* was not included in the analysis due to the highly specific expression pattern restricted to the brain. N = 4 for each sample. Data shown as boxplots. Individual points represent relative expression (housekeeping gene used for normalization: *actb1 (β-actin*)). Measurements with SD >2 from the mean expression value were considered outliers.
(PDF)

S7 Fig *rprma* and *rprmb* are expressed during zebrafish angiogenesis. (A-B) Lateral views of whole-mount in situ hybridization at 48hpf. *rprma/b* are expressed in the mesencephalic vein (MsV), the dorsal longitudinal vein (DLV), the primordial hindbrain channel (PHBC), the primary head sinus (PHS), the nasal ciliary artery (NCA), the primary head sinus (PHS) and inner optic circle (IOC). Inset magnification shows that *rprma/b* are expressed dispersedly throughout the head vessels. (A’-B’) Lateral views of the trunk vasculature, where *rprma/b* are expressed in hypochord (Hp, black arrow), the posterior cardinal vein (PCV, white bracket) and the intersegmental vessels (ISV, white arrows).
(PDF)

S8 Fig FISH to localize *rprm* gene expression in zebrafish larvae. Top panels showing head or trunk views. (A-B) Vascular-specific transgenic *Tg(fli1a*:*EGFP)* marks endothelial cells and is revealed with the anti-GFP antibody. (C-F) FISH to detect *rprma*, *rprml* and *pdgfrβ* genes expression. *pdgfrβ* is used as mural cell marker. (G-H) merged confocal imaging.
(PDF)

S1 Table Differentiating elements between RPRM protein sequences. Shown are *RPRM*-, *RPRML*- and *RPRM3*-specific amino acid substitutions and positions, relative to the methionine (M) at position 1 at the N-terminus of the proteins, which are colored in Fig 2.
(PDF)

S2 Table Raw data from RT-qPCR in adult zebrafish brain and gut. (TXT)
