# Supplementary figures and images for: Reprimo tissue-specific expression pattern is conserved between zebrafish and human
Source: PLoS One. 2017 May 31;12(5):e0178274. doi: 10.1371/journal.pone.0178274 (PMC5451059; doi:10.1371/journal.pone.0178274)

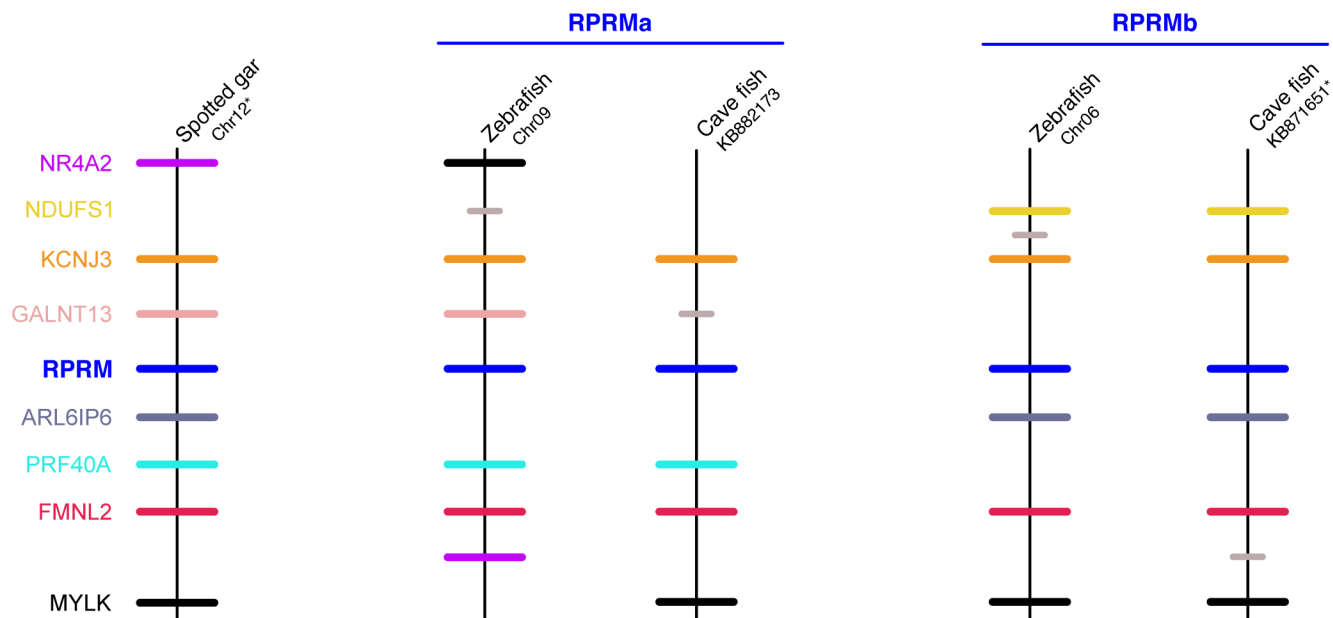

Figure. S1

Supplement: S1 Fig — (PDF) [file pone.0178274.s001.pdf]

Relative expression curves of all RPRM genes by hours post-fertilization

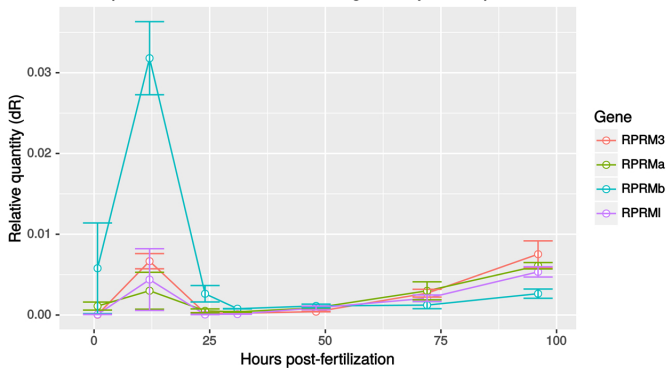

Figure. S2

Supplement: S2 Fig — Relative mRNA expression of rprma, rprmb and rprml are plotted across indicated developmental points. Each point represents the average measurement of three biological replicates. Bars correspond to standard error of the mean (SEM). Hpf: hours post-fertilization. Relative expression was normalized against actin, beta 1 (actb1). (PDF) [file pone.0178274.s002.pdf]

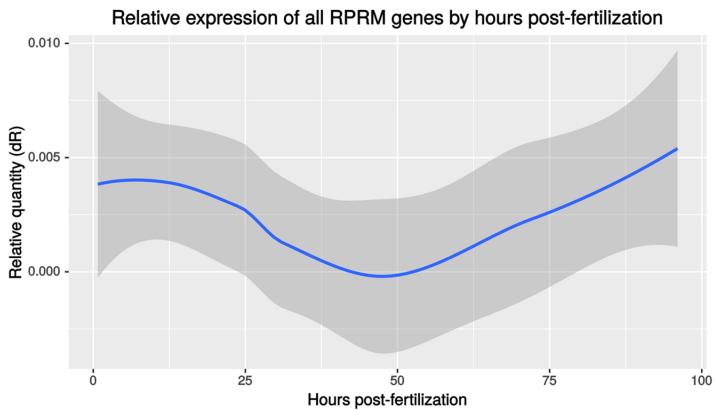

Figure. S3

Supplement: S3 Fig — Relative mRNA expression of rprm genes are plotted across indicated developmental points. Each point represents the average measurement of the relative expression of the 4 rprm genes (rprma/rprmb, rprml and rprm3). The dark grey area around the curve represents SEM. (PDF) [file pone.0178274.s003.pdf]

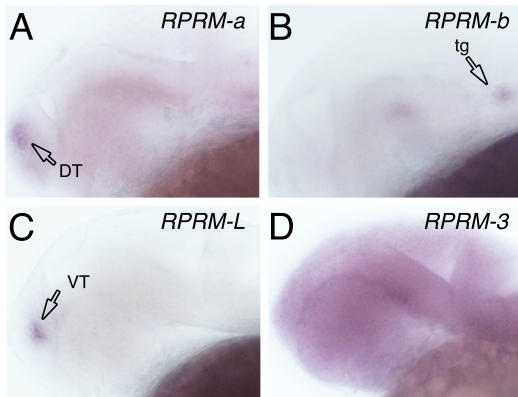

Figure. S4

Supplement: S4 Fig — rprm expression patterns were examined using whole-mount in situ hybridization in wild-type embryos at (A-D) 1 day post-fertilization [hpf]. (A-D) Lateral views. (A-C) At these developmental stages, rprma, rprmb and rprml transcripts are located in neuronal populations such as dorsal thalamus (DT), ventral thalamus (VT) and the cranial placode in the tigreminal ganglia (tg). (D) rprm3 is ubiquitously expressed through the brain. (PDF) [file pone.0178274.s004.pdf]

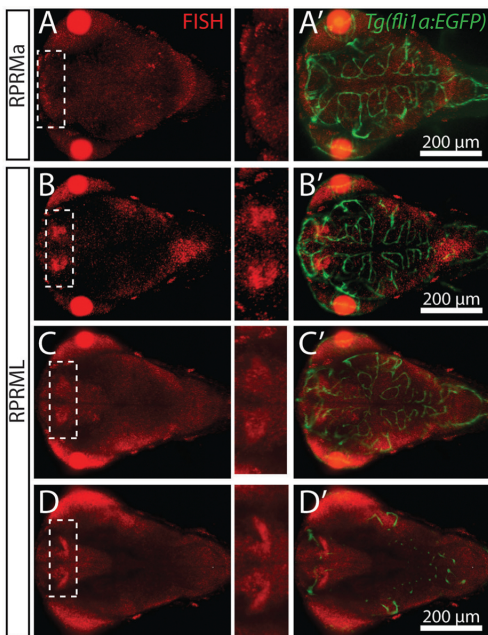

Figure. S5

Supplement: S5 Fig — (A-D) rprma and rprml expression was detected by whole-mount fluorescent in situ hybridization (FISH) at 72hours post-fertilization [hpf]. (A) Confocal cropping shows rprma expression in the anterior neurons of the telencephalon. (C-D) Confocal sectioning shows rprml expression in the posterior neurons within the forebrain (inset magnification). (A’D’) Transgenic Tg(fli1a:EGFP) is expressed in the endothelial cells within the major blood vessels of the head. (PDF) [file pone.0178274.s005.pdf]

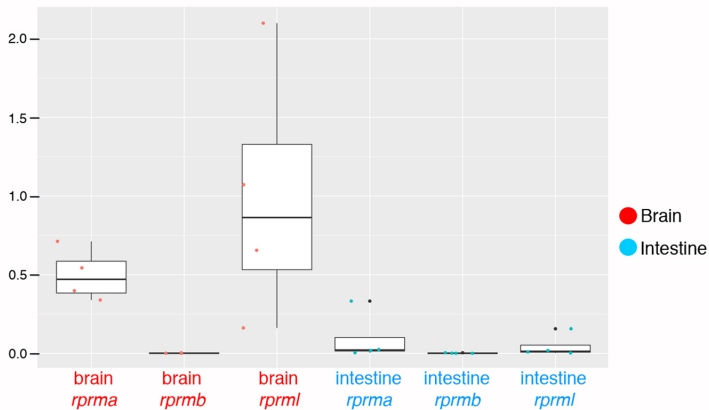

Figure. S6

Supplement: S6 Fig — rprm genes are expressed in the brain (red dots). rprml and rprma are the highest expressed genes in the brain (red dots) and intestine (blue dots), respectively. rprmb has not significant expression in either tissue. rprm3 was not included in the analysis due to the highly specific expression pattern restricted to the brain. N = 4 for each sample. Data shown as boxplots. Individual points represent relative expression (housekeeping gene used for normalization: actb1 (β-actin)). Measurements with SD >2 from the mean expression value were considered outliers. (PDF) [file pone.0178274.s006.pdf]

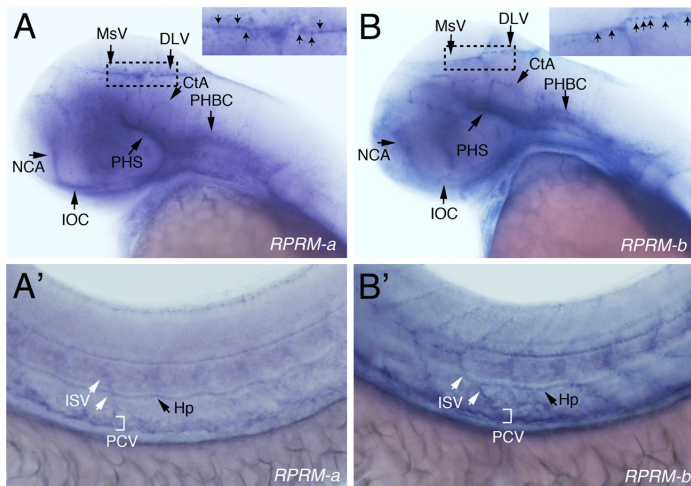

Figure S7

Supplement: S7 Fig — (A-B) Lateral views of whole-mount in situ hybridization at 48hpf. rprma/b are expressed in the mesencephalic vein (MsV), the dorsal longitudinal vein (DLV), the primordial hindbrain channel (PHBC), the primary head sinus (PHS), the nasal ciliary artery (NCA), the primary head sinus (PHS) and inner optic circle (IOC). Inset magnification shows that rprma/b are expressed dispersedly throughout the head vessels. (A’-B’) Lateral views of the trunk vasculature, where rprma/b are expressed in hypochord (Hp, black arrow), the posterior cardinal vein (PCV, white bracket) and the intersegmental vessels (ISV, white arrows). (PDF) [file pone.0178274.s007.pdf]

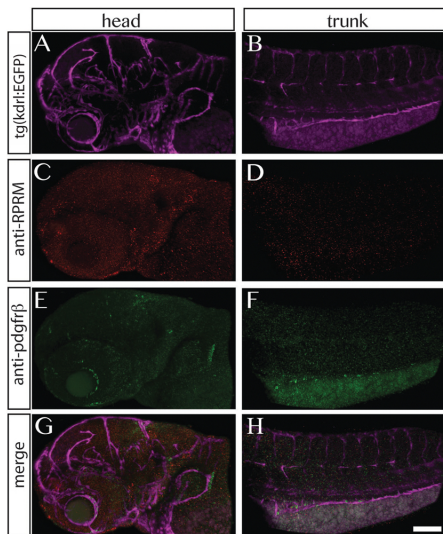

Figure S8

Supplement: S8 Fig — Top panels showing head or trunk views. (A-B) Vascular-specific transgenic Tg(fli1a:EGFP) marks endothelial cells and is revealed with the anti-GFP antibody. (C-F) FISH to detect rprma, rprml and pdgfrβ genes expression. pdgfrβ is used as mural cell marker. (G-H) merged confocal imaging. (PDF) [file pone.0178274.s008.pdf]
