# Supplementary material for: Reprimo tissue-specific expression pattern is conserved between zebrafish and human
Source: PLoS One. 2017 May 31;12(5):e0178274. doi: 10.1371/journal.pone.0178274 (PMC5451059; doi:10.1371/journal.pone.0178274)
Supplement: S1 Table — Shown are RPRM-, RPRML- and RPRM3-specific amino acid substitutions and positions, relative to the methionine (M) at position 1 at the N-terminus of the proteins, which are colored in Fig 2. (PDF) [file pone.0178274.s009.pdf]

Table 1. Differentiating elements between RPRM protein sequences

Species	Gene	Aminoacid-Position
Human	RPRM	D-10, M-62, G-73
Mouse	RPRM	D-10, M-62, G-73
Zebrafish	RPRMa	D-09, M-56, G-67
Zebrafish	RPRMb	D-10, M-55, G-66
Zebrafish	RPRM3	D-20, G-37, I-65, L-85, I-99
Human	RPRML	F-5, G-33, G-42, L-119
Mouse	RPRML	F-5, G-30, G-39, L-116
Zebrafish	RPRML	F-5, G-25, G-35, L-113

Shown are RPRM-, RPRML- and RPRM3-specific amino acid substitutions and positions, relative to the methionine (M) at position 1 at the N-terminus of the proteins, which are colored in Fig 2.
